# Secure attachment to caregiver prevents adult depressive symptoms in a sex-dependent manner: A translational study

**DOI:** 10.1016/j.isci.2024.111328

**Published:** 2024-11-21

**Authors:** Camilla Mancini, Lucy Babicola, Gilda Chila, Matteo Di Segni, Diana Municchi, Sebastian Luca D’Addario, Elena Spoleti, Alice Passeri, Carlo Cifani, Diego Andolina, Simona Cabib, Fabio Ferlazzo, Marco Iosa, Rodolfo Rossi, Giorgio Di Lorenzo, Massimiliano Renzi, Rossella Ventura

**Affiliations:** 1 University of Camerino, School of Pharmacy, Pharmacology Unit, Camerino, Italy; 2 IRCCS Santa Lucia Foundation, Rome, Italy; 3 Department of Physiology and Pharmacology, Sapienza University, Rome, Italy; 4 Child Psychopathology Unit, Scientific Institute IRCCS Eugenio Medea, Bosisio Parini, Italy; 5 Department of Psychology, Sapienza University, Rome, Italy; 6 Department of Systems Medicine, Tor Vergata University of Rome, Rome, Italy; 7 IRCCS San Raffaele, Rome, Italy

**Keywords:** biological sciences, neuroscience, behavioral neuroscience

## Abstract

Although clinically relevant, evidence for a protective effect of early secure attachment against the development of depressive symptoms in adulthood is still inconsistent. This study used a translational approach to overcome this limitation. The analysis of a non-clinical adult population revealed a moderating effect of secure attachment on depressive symptoms in women only. Thus, we tested the causal link between early attachment with caregiver and adult depressive-like phenotypes in a mouse model of early adversities that is especially effective in females. Repeated cross fostering (RCF) in the first postnatal days prevented the development of pups’ secure attachment with the caregiver as tested in a rodent version of the “strange situation”—the standard human test—induced depressive-like behaviors and altered activity of the ventral tegmental area dopamine neurons in adulthood. Finally, a stable alternative caregiver during the RCF experience prevented all these effects, modeling human “earned attachment.”

## Highlights

• Secure attachment prevents vulnerability to depressive symptoms in women
• RCF undermines pups' secure attachment and induces depressive-like behavior
• RCF alters the activity of the ventral tegmental area dopamine neurons in adulthood
• A stable alternative caregiver during RCF prevents these negative outcomes

## Graphical abstract

**Figure:**
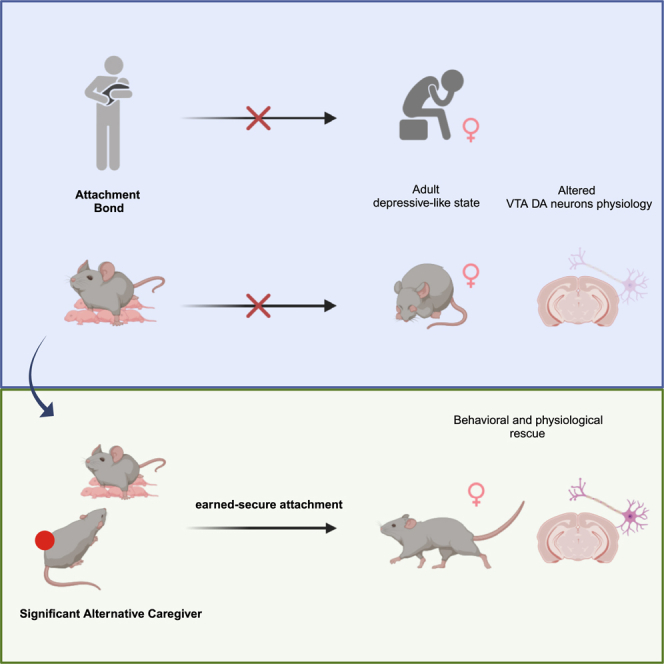

## Introduction

The early infant-caregiver relationship in mammals is paramount for emotional development and psychological well-being.^1^^,^^2^ Indeed, a functional dyadic interaction establishes a “secure” attachment bond,^1^^,^^3^ which, in turn, shapes the organization of the brain by guiding synaptic connectivity.^4^ By contrast, a dysfunctional parent-child interaction fosters insecure attachment, which increases vulnerability to psychopathology, notably depression, in adulthood.^1^^,^^5^^,^^6^^,^^7^^,^^8^^,^^9^^,^^10^ Thus, unveiling the psychobiological pathway(s) linking early insecure attachment to adult mental disturbances has a relevant clinical value. Nonetheless, human findings supporting the link between attachment and depression are still inconsistent.^5^

Interestingly, early attachment is a phylogenetically preserved behavioral system with striking similarities among altricial species,^11^^,^^12^ and this feature makes it particularly well suited for cross-species studies.^2^^,^^7^ Thus, the use of animal models is key to investigating translationally the causal links between early attachment and dysfunctional phenotypes expressed in adulthood.^5^^,^^13^ In fact, this experimental approach allows both the manipulation of relevant independent variables (such as genotype, sex, and life experience) within specific time windows and the direct analysis of the underlying neurophysiological processes.^14^^,^^15^ The repeated cross fostering (RCF), an experimental paradigm recapitulating early interference with a stable mother-pup attachment bond,^16^^,^^17^^,^^18^^,^^19^ has been proposed to model in mice the unstable early environment. Indeed, by adopting the RCF model, we showed that exposing pups to an unstable early environment within the first few days after birth affects the mesocorticolimbic dopaminergic (DA) transmission^15^^,^^19^^,^^20^^,^^21^^,^^22^ and, particularly, the ventral tegmental area (VTA)-related behavioral and transcriptomic pattern in mice, in a sex- and strain-dependent manner.^22^ At the neurophysiological level, we reported that the RCF paradigm affects the hyperpolarization-activated current (I_h_ current) of the VTA DA neurons in resilient to depression females of the C57BL/6J strain.^23^ Interestingly, we have also demonstrated that RCF can cause an opposite behavioral modulation (that is, increased vulnerability to depression-like phenotype) in female mice with a different genetic background, the DBA2/J (DBA) strain.^19^^,^^22^^,^^24^ Thus, the RCF seems to affect the psychobiological pathway(s) of mice differently depending on their sex and/or genetic background. This reflects what is reported in humans, where early environmental factors (e.g., childhood adversities) can influence adult behavioral outcomes and epigenetic marks in a genotype-^25^ and sex-related^26^ manner.^27^ Thus, the RCF paradigm can reliably model the early environment × genotype × sex interaction observed in humans.^28^

A step further from this, in humans, the availability of a significant alternative caregiving figure may prevent the negative consequences of an insecure attachment bond, a phenomenon known as “earned security.”^8^^,^^9^ However, human studies are, to date, mainly correlational, and our knowledge of earned security suffers from a lack of direct investigation of the underpinning neurobiological pathways. Here, using a translational approach, we aimed to help fill this gap and propose a rodent model of “earned attachment” to promote secure attachment in mice. Thus, first, we investigated the link between attachment style and vulnerability to depressive symptoms in a human non-clinical cohort and found that secure attachment can prevent depressive symptoms in women. Second, using the RCF paradigm, we evaluated the consequences of an unstable early environment in DBA2/J (DBA) mice and found that RCF both impaired mouse attachment-like behavior in young animals and induced depressive-like behavior and alteration of VTA DA neurons in adult females. And last, we showed that the presence of a significant alternative caregiver (SAC, a virgin female dam) during RCF procedure plays a protective role both in the short-term, promoting secure attachment in pups, and in the long-term by preventing both behavioral and neurophysiological alterations in adult female mice.

## Results

### Human study

#### Secure attachment protects against depression symptoms vulnerability in women from a non-clinical population

**Figure 1:**
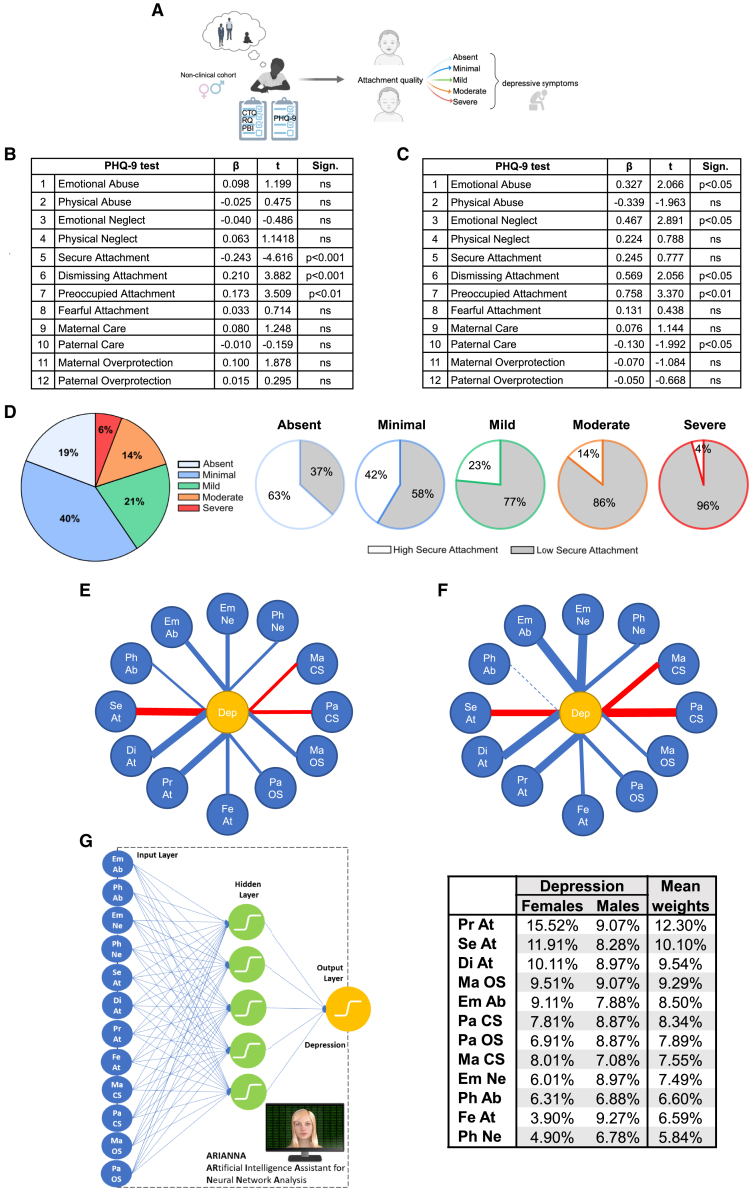
Secure attachment protects against vulnerability to depression symptoms in women from a non-clinical population (A) Schematics depicting the experimental paradigm adopted on non-clinical cohorts. (B and C) Regression analysis of PHQ-9 evaluations in women (B) and men (C); note that in women, the secure attachment acts as a protective factor against depressive symptoms vulnerability. (D) Relationship between depressive symptoms severity (PHQ-9 score; left) and secure attachment level (high, low; right) in non-clinical women. The proportion of women with high secure attachment decreased across groups with depression symptoms of increasing severity (PHQ-9 score). As shown (D, left) in women, severe depression symptoms were evident in 6% of the sample, moderate in 14%, mild in 21%, minimal in 40%, and absent in 19% of the cohort and, intriguingly, the proportion of women with high secure attachment score decreased across these groups representing the 63% of individuals with depressive symptoms absent; 42% with minimal; 23% with mild; 13% with moderate, and 4% with severe depression symptoms (D, right). (E and F) Correlation nodes analysis for women (E) and men (F) (Dep, depression; Ph Ab, physical abuse; Em Ab, emotional abuse; Em Ne, emotional neglect; Ph Ne, physical neglect; Ma CS, maternal care; Pa CS, paternal care; Ma OS, maternal overprotection; Pa OS, paternal overprotection; Fe At, fearful attachment; Pr At, preoccupied attachment; Di At, dismissing attachment; Se At, secure attachment). (G) Artificial intelligence assistant for neural network (ARIANNA) estimates of % weight associated with each independent variable to predict the dependent variable (PHQ-9 score). Both correlation nodes analysis and ARIANNA confirmed regression analysis.

For the present study, we chose a nonclinical population to evaluate the link between different forms of human insecure attachment and the expression of depressive symptoms in adulthood. To do this, we collected a set of surveys (see STAR Methods for details) from 512 individuals at least 18 years old (398 women, aged on average 25.54 ± 0.03 years; 114 men aged, on average, 26.14 ± 0.06 years) bearing non-clinical depressive symptoms. Primarily, surveys aimed at disclosing the participants’ childhood experience (scored retrospectively); thus, we used the Parental Bonding Inventory (PBI)^29^ and the Relationship Questionnaire (RQ)^30^ to assess the perceived parental care and attachment style. Further, we used the Childhood Trauma Questionnaire (CTQ)^31^ to determine exposure to childhood trauma. Finally, the vulnerability to depressive symptoms was measured by the widely used Patient Health Questionnaire-9 (PHQ-9).^32^ We checked for possible differences between women and men on the sub-scales of PBI, RQ, CTQ, and PHQ-9 using a multivariate analysis of variance on the dataset as a whole (Figure 1A; Table S1 in the supplementary information): our analysis showed a significant effect of gender (Wilks’ Lambda = 0.962, F(10,498) = 1.991; *p* < 0.05). Also, the following univariate analyses showed significant effects of gender for both maternal care (*p* < 0.05) and paternal care (*p* < 0.05), with men showing higher scores than women. Notably, although not significant, we also found a strong effect for PHQ-9 (*p* = 0.094), with women showing higher scores for depressive symptoms compared to men (Table S1). These findings suggested a possible gender-related divergence of vulnerability to depressive symptoms.^33^ We thus performed multiple linear regression analyses of women and men datasets separately to ascertain if vulnerability to depression symptoms was linked to childhood trauma, parental bonding, or attachment style in a gender-dependent manner. With regards to childhood trauma (typically a strong risk factor for depression), we entered the CTQ *total* score and the *single* CTQ sub-scale scores in separate regression analyses to investigate the relative relevance of each different early aversive experience (sexual abuse sub-scale was excluded from the analysis due to low frequency in the enrolled sample). The analysis of CTQ total score returned high significance for depressive symptoms both for women (F(9,382) = 22.119, *p* < 0.0001) and men (F(9,101) = 10.636, *p* < 0.001), explaining respectively 32% and 43% of the variance (adjusted R^2^). Similar results were obtained with CTQ sub-scales scores both for women (F(12,382) = 16.877, *p* < 0.0001) and men (F(12,101) = 11.022, *p* < 0.001), explaining respectively 33% and 52% of the variance (adjusted R^2^). Dismissing and preoccupied attachment styles emerged as significant predictors of depressive symptoms vulnerability for women (Figure 1B). In contrast, dismissing and preoccupied attachment, as well as emotional neglect and emotional abuse, emerged as significant predictors for men (Figure 1C) while secure attachment and paternal care emerged as protective factors for women and men, respectively (Figures 1B and 1C). Of note, trauma did not emerge as a significant predictor for depressive symptoms vulnerability in women or men (Tables S2 and S3).

Altogether, our data confirm the association between insecure attachment and depressive symptoms vulnerability in humans.^5^ Further to this, present data interestingly support the pivotal role played by secure attachment as a protective factor for mental health,^3^^,^^5^^,^^8^^,^^9^ especially in women. However, it might be observed that the sample size was smaller for male than for female participants, making it difficult to compare the generalizability of the results for the two samples.

Such a link between depression symptoms vulnerability and secure attachment style in women appeared even more evident by re-sorting the sample individuals according to the quality of their secure attachment (high or low) and their PHQ-9 score (from absent to severe depression symptoms; see STAR Methods for details). Importantly, this approach indicated that lower depressive symptoms were associated with higher scores of secure attachment and, vice versa, higher depressive symptoms matched lower scores of secure attachment (Figure 1D). Thus, in women, severe depression symptoms were evident in 6% of the sample, moderate in 14%, mild in 21%, minimal in 40%, and absent in 19% of the cohort (Figure 1D left) and, intriguingly, the proportion of women with high secure attachment score decreased across these groups representing the 63% of individuals with depressive symptoms scored “absent”; 42% with “minimal”; 23% with “mild”; 14% with “moderate”; and 4% with “severe” depression symptoms (Figure 1D, right; Figure S1).

We confirmed our observations on humans by adopting two more analytical approaches: the analysis of correlation nodes (Figures 1E and 1F) and the predictions exploited by an artificial neural network (Figure 1G). First, the analysis of correlation nodes, depicted in Figures 1E and 1F, confirmed that, for women, among all the variables considered for the participants’ childhood experience, secure attachment returned the strongest negative correlation with depression symptoms (R = – 0.47, *p* < 0.001) (Figure 1E). Instead, dismissing attachment (R = 0.45, *p* < 0.001) and preoccupied attachment (R = 0.42, *p* < 0.001) were strongly positively correlated with depression symptoms (Figure 1E), and, in men, paternal care was negatively correlated with depression symptoms (R = – 0.53, *p* < 0.001). This confirmation was described previously. Last, when an artificial neural network (Figure 1G; see STAR Methods and Table S6 for details) was implemented to investigate the relevance of each of the childhood variables to depressive symptoms, the factors with the higher weights on depression resulted to be preoccupied attachment (12.30%), insecure attachment (10.10%) and dismissing attachment (9.54%), with weights higher for females compared to males (on average: +10.68%). These findings substantially confirmed our regression analysis results: for women, the most influential variables related to attachment (with preoccupied, insecure, and dismissing attachments as the only statistically significant predictors identified by regression analysis for females).

Overall, our variance, regression, sorting, correlational, and AI analyses of human data point to the secure attachment as the most relevant protective factor against vulnerability to depression symptoms in a non-clinical women population.

### Animal study

#### RCF The presence of a SAC during the protocol prevents insecure attachment in female mice and mimics the protective effects of an alternative supportive figure in humans

**Figure 2:**
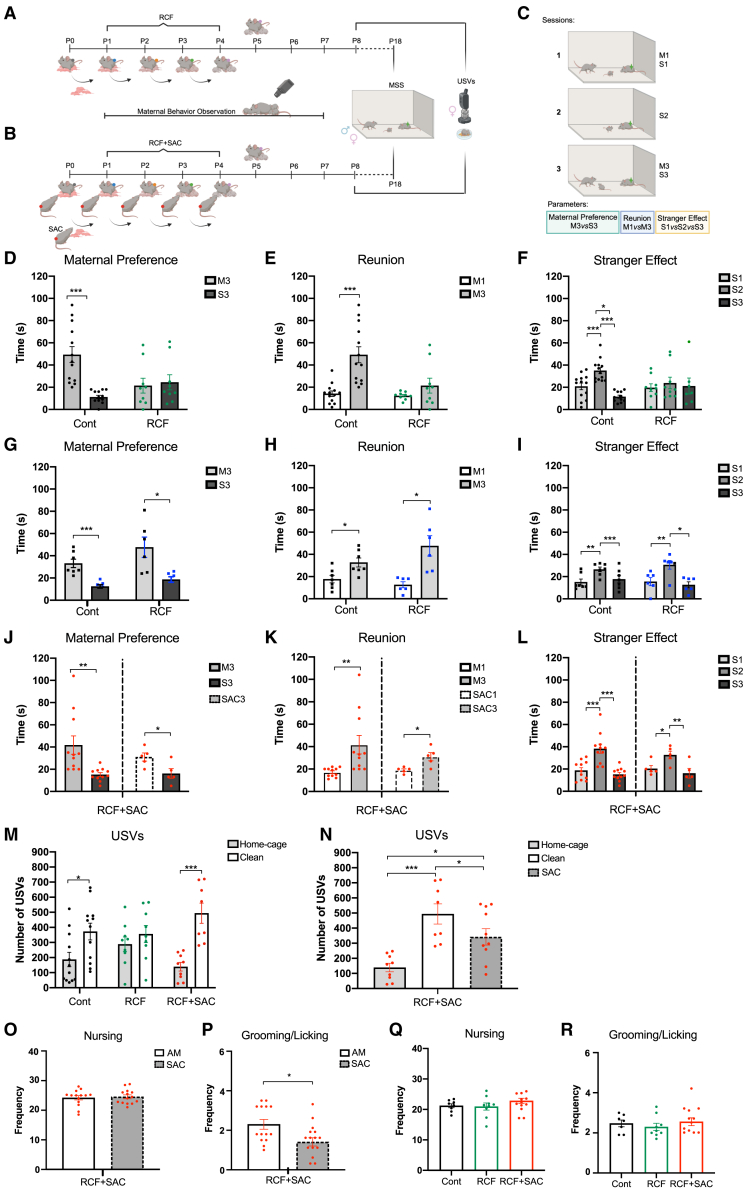
A significant alternative caregiver plays a protective role during mouse development by inducing an earned-secure attachment (A–C) Schematics depicting the experimental paradigm adopted on mice along development, including (A) repeated cross fostering protocol (RCF), (B) RCF plus a significant attachment caregiver (RCF+SAC), and (C) strange situation paradigm (MSS). (D–I) MSS parameters were used to evaluate the attachment-like style in DBA female pups (D–F) and males (G–I). Note that in DBA females (but not in males), RCF significantly impaired the attachment-like behavior. (J–L) MSS parameters for RCF+SAC. The left-hand halves of the panels depict the effect of SAC on the attachment-like style of RCF female pups (RCF+SAC) with the adoptive mother (AM). The right-hand halves of the panels depict the effect of SAC on the attachment-like style of RCF female pups (RCF+SAC) with the SAC. Note that the presence of a SAC rescued the impaired attachment-like behavior of RCF females with the AM. Also, note that RCF+SAC pups earned a stable attachment with the SAC. (M and N) Ultrasonic vocalization calls (USVs) emitted by Cont, RCF, and RCF+SAC female pups in the home-cage or clean-cage (M) and when exposed to SAC bedding material (N). In both conditions, the SAC rescued the behavior shown by RCF females. (O and P) Nursing (O) and grooming/licking (P) behaviors shown by the AM and SAC during the first four days (P1–P4) of co-caring. (Q and R) Like (O and P) for the “mother” (biological for the Cont mice and adoptive for RCF and RCF+SAC mice) during the first seven days (P1–P7). Statistical significance denoted with: ∗*p* < 0.05; ∗∗*p* < 0.01; ∗∗∗*p* < 0.001.

To lay robust bases for an experimental paradigm recapitulating in mice the role and effects of a SAC as observed in humans, firstly, we capitalized on our well-validated model of early adversity (RCF; Figure 2A).^14^^,^^15^^,^^16^^,^^19^^,^^21^^,^^22^^,^^23^ We directly investigated the attachment-like bond in DBA females and males and evaluated how early experiences may disrupt the attachment bond in rodents. In particular, since in humans, the evaluation of the attachment style of young children in experimental settings is commonly performed using the strange situation procedure (SSP),^10^^,^^34^ here we adopted the mouse strange situation (MSS) test, an experimental paradigm with translational validity developed to match the SSP^35^ (Figure 2C for schematic representation), and quantified common features of secure attachment (see STAR Methods for details), as: “Maternal Preference” (i.e., M3*vs*S3), “Reunion” (i.e., M3*vs*M1), and “Stranger Effect” (i.e., S1*vs*S2*vs*S3).

The ANOVA analysis of the duration of each of these behavioral patterns for RCF and Cont females returned a significative interaction between “treatment” (RCF or Cont) and each of the indexes studied: “Maternal Preference” (F(1,20) = 9,023; *p* < 0.01; Figure 2D), “Reunion” (F(1,20) = 7.802; *p* < 0.05; Figure 2E), and “Stranger Effect” (F(2,40) = 10.069; *p* < 0.001; Figure 2F).

Thus, while Cont females showed significant “Maternal Preference” (M3>S3; *p* < 0.001), “Reunion” (M3>M1; *p* < 0.001), and “Stranger Effect” (S1<S2>S3; *p* < 0.001), each of these parameters was not significant in RCF females exposed to the last adoptive mother (AM) (Figures 2D–2F).

Further, in line with our previous reports showing a lack of long-term effects in RCF DBA males on depressive-like behavior (i.e., forced swimming test [FST] and tail suspension test [TST]),^22^ the attachment-like style was found to be unaltered in RCF vs*.* Cont males for all the parameters investigated: “Maternal Preference” (M3>S3; Cont: F(1,12) = 25.433; *p* < 0.001; RCF: F(1,10) = 9.321; *p* < 0.05; Figure 2G); “Reunion” (M3>M1; Cont: F(1,12) = 9.086; *p* < 0.05; RCF: F(1,10) = 13.429; *p* < 0.005; Figure 2H); and, “Stranger Effect” (S1<S2>S3; Cont: F(2,18) = 12.066; *p* < 0.001; RCF: F(2,15) = 5.475; *p* < 0.05; Figure 2I). These results confirmed that RCF interferes with the attachment-like style in DBA mice in a sex-dependent manner by affecting females only. Next, we tested whether the presence of a SAC (Figure 2B) could “rescue” the secure attachment-like style impaired in RCF females. Notably, the ANOVA analysis of our results clearly showed that the secure attachment-like style in RCF+SAC animals was similar to Cont mice, supporting the rescue effects induced by the SAC: “Maternal Preference” (M3>S3) (F(1,20) = 9.755; *p* < 0.01; Figure 2J); “Reunion” (M3>M1) (F(1,20) = 8.523; *p* < 0.01; Figure 2K); and “Stranger Effect” (S1<S2>S3) (F(2,30) = 17.440; *p* < 0.001; Figure 2L), when RCF+SAC pups were tested in presence of the last AM. In addition, when we tested the attachment-like style of the RCF+SAC pups in presence of the SAC (instead of the last AM), we also observed a stable attachment-like style: “Maternal Preference” (SAC3>S3; F(1,8) = 6.836; *p* < 0.05; Figure 2J); “Reunion” (SAC3>SAC1; F(1,8) = 9.730; *p* < 0.05; Figure 2K) and “Stranger Effect” (S1<S2>S3; F(2,12) = 5.676; *p* < 0.05; Figure 2L). These data (resumed in Table S4) indicate the protective role of the SAC in preventing the impairment of the attachment bond with the last AM otherwise induced by RCF and, importantly, strongly support the establishment of an earned-secure attachment.

To further investigate the protective effects associated with the presence of the SAC, we also evaluated the parameter “separation anxiety” by analyzing the ultrasonic vocalization calls (USVs) emitted by Cont, RCF, and RCF+SAC female pups when exposed to home-cage bedding material or bedding materials without familiar cues (“clean condition”).^16^^,^^22^ The analysis of the USVs showed a significant “treatment”×“bedding” interaction (F(2,53) = 3.395; *p* < 0.05) and, in line with our previous reports,^21^^,^^22^ while Cont pups exhibited more USVs in clean bedding vs. home-cage (*p* < 0.05) indicative of reduced anxiety when in presence of the nest odor, RCF pups showed no significant difference across conditions (Figure 2M), confirming the more substantial separation anxiety induced by the interference with the attachment bond.^17^^,^^22^ Notably, the presence of the SAC rescued the calming effect induced by the home-cage bedding material (*p* < 0.0001; Figure 2M) in RCF females. Thus, like Cont, RCF+SAC females vocalized less when exposed to home-cage bedding material compared to the odorless, clean one. Moreover, when exposed to the SAC bedding, RCF+SAC pups vocalized more than in the home-cage nest’s scent (*p* < 0.05) but less than in the clean, odorless bedding (*p* < 0.05; Figure 2N) supporting the protective role of the SAC (F(2.24) = 11.216; *p* < 0.001).

#### A SAC does not affect maternal behavior

Unlike other early adversities models, RCF does not modify maternal behavior.^16^^,^^19^ However, to assess if the maternal behavior of AMs was affected by the SAC during the four days of co-caring (P1–P4), we investigated the maternal behavior (nursing, grooming/licking) of both the AM and the SAC. There was no significant difference for nursing between AM and SAC (t = 0.353, *p* = n.s.; Figure 2O), while a significant difference was found for the grooming/licking behaviors, with AM showing higher frequency than SAC (t = 2.729; *p* < 0.05; Figure 2P). Moreover, in line with previously published data,^16^^,^^17^^,^^19^ when analyzing the maternal behavior of the AM (or the biological mother in the Cont group) from P1 to P7, we did not observe any difference between Cont and RCF or between RCF+SAC and the other experimental groups for both nursing (F(2,28) = 0.817; *p* = n.s.; Figure 2Q) and grooming/licking (F(2,28) = 0.755; *p* = n.s.; Figure 2R).

#### A Significant Alternative Caregiver prevents the adult depression-like phenotype

**Figure 3:**
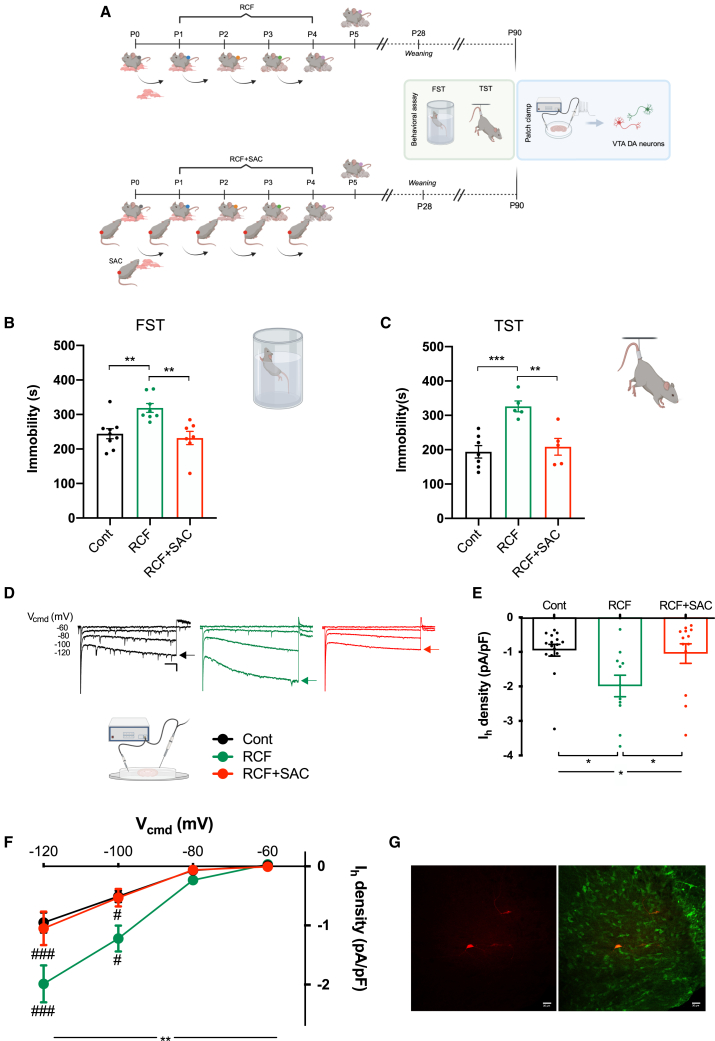
The presence of a significant alternative caregiver prevents depression-like phenotype and VTA DA neurons physiological alterations in adult RCF females (A) Schematics depicting the experimental paradigm adopted on adult mice. (B and C) Data from forced swimming test (FST; B) and tail suspension test (TST; C). Note that RCF mice showed increased immobility compared to Cont mice in both FST and TST, and this response was prevented by the presence of a SAC. (D–G) Panels depicting patch-clamp investigations. (D), typical traces for I_h_ currents elicited in iVTA DA neurons by V_cmd_ steps (shown; scale bar: 100 ms; 50 pA); the maximal, average I_h_ amplitude was estimated at the current steady-state (arrows) for each step; (E), pooled data for I_h_ current density (calculated at V_cmd_ −120 mV); (F), mean full I-V relationships for I_h_ current density; and (G), typical immunostaining showing that the patched cell (red; left panel) was a TH^+^ DA neuron (green) within the iVTA (right panel). Recorded neurons were considered for analysis only if TH^+^ (orange; right panel); scale bar: 30 μm. Note that RCF iVTA DA neurons had increased I_h_ current compared to Cont, which was shown both as increased current density and larger I-V mean curves, and that the presence of a SAC prevented this modulation. Statistical significance denoted with: ∗*p* < 0.05; ∗∗*p* < 0.01; ∗∗∗*p* < 0.001, #*p* < 0.05, ###*p* < 0.001.

To test whether the SAC prevented the development of the depression-like phenotype observed in adult DBA RCF females,^22^^,^^33^ we compared DBA females from RCF, RCF+SAC and Cont groups using the FST and TST, two behavioral tests commonly used to evaluate the depressive-like phenotype in rodents (Figure 3A). Moreover, the open field test (OF) was performed to assess locomotor activity and anxiety behavior^22^ (Figure S2). The ANOVA analysis showed a significant “treatment” effect for both FST (F(2,21) = 9.055; *p* < 0.01) and TST (F(2,14) = 12.948; *p* < 0.001). As previously reported,^19^^,^^22^ RCF females showed increased immobility compared to Cont in both FST (*p* < 0.01; Figure 3B) and TST (*p* < 0.001; Figure 3C). Notably, RCF+SAC mice showed decreased immobility in both tests compared to RCF (FST: *p* < 0.01; TST: *p* < 0.01), and matched Cont levels (Figures 3B and 3C). No difference was observed across groups for locomotor activity nor anxiety-like behavior in the OF (locomotor activity: F(1,19) = 0.04; *p* = n.s.; anxiety behavior: (F(2,19) = 2.577; *p* = n.s.; Figure S1). Together, these results confirmed the role of the SAC in preventing the development of depression-like phenotype in adulthood, in agreement with the protective role of the earned-secure attachment on depression reported in humans.^8^^,^^9^

#### A SAC rescues the long-lasting alteration of I_h_ current in VTA DA neurons of RCF females

Having supported the validity of our animal model at the behavioral level, we aimed to identify which possible mediator(s) could link dysfunctional attachment-like style and depression-like behavior in adults at the cellular level. To do this, first, we investigated in adult DBA females the long-term effects of RCF on the physiological properties of DA neurons within the intermediate VTA (iVTA); secondly, we asked whether a SAC could interfere with RCF effects. Based on our previous report linking decreased I_h_ current in iVTA DA neurons with the resilience to depression-like phenotype in C57 adult females,^23^ here we hypothesized that the RCF-dependent depression-like phenotype found in DBA females could relate to a long-lasting increase of I_h_ currents in these neurons. To test our hypothesis, we performed voltage-clamp experiments on DA (TH^+^) neurons of the iVTA (Figure 3G) from Cont, RCF, and RCF+SAC DBA females. In particular, (1) we estimated the neuronal maximal I_h_ current density (at V_cmd_ −120 mV; Figures 3D and 3E) and (2) we described “I_h_ current density (I)-membrane voltage (V)” relationship (Figure 3F). First, in line with our hypothesis we found that indeed iVTA neurons were affected by RCF in DBA females as they had significantly larger I_h_ current density compared to Cont (Figures 3D and 3E). Also, and notably, such potentiation was completely abolished in animals exposed to RCF in the presence of the SAC (Figures 3D and 3E). In details, for current density: Cont −1.0 ± 0.2 pA/pF (16 neurons/7 mice); RCF –2.0 ± 0.3 pA/pF (11 neurons/8 mice); and RCF+SAC –1.0 ± 0.3 pA/pF (13 neurons/5 mice); Kruskal-Wallis test: *p* = 0.0120, with Dunn’s multiple comparison: *p* < 0.05 for both RCF vs. Cont and RCF+SAC vs. RCF; for RCF+SAC vs. Cont, *p* > 0.9999. Similar to what found for I_h_ maximal density, the I-V relationship for RCF iVTA DA neurons was significantly larger compared to that of both Cont and RCF+SAC neurons (Figure 3F). In detail, the 2-way RM ANOVA analysis returned for “*in vivo* treatment” (F(2,37) = 5.665, *p* < 0.01) with significant interaction between “*in vivo* treatment” and “V_cmd_” (F(6,111) = 4.921, *p* = 0.001) leading to the Bonferroni’s *post-hoc* analysis that returned a significative difference: for V_cmd_ −100 mV, RCF vs. Cont and RCF+SAC vs. RCF (both *p* < 0.05); and for V_cmd_ −120 mV, RCF vs. Cont (*p* < 0.0001) and RCF+SAC vs. RCF (*p* < 0.001). Of note, exposure to RCF did not alter the intrinsic, sub-threshold properties nor the spontaneous or evoked excitability (respectively, spontaneous firing and rheobase) of the iVTA DA neurons (Table S5). Thus, our voltage-clamp results indicate that, in our animal model, the modulation of the I_h_ current in iVTA DA neurons might represent one of the possible candidates to link early dysfunctional attachment to depression in adulthood.

## Discussion

Here, by implementing a mouse model of early interference with the attachment bond (RCF) we report strong evidence for the protective effect of secure attachment with a caregiver against the development of depressive phenotypes in adulthood. Our findings translational relevance to the experimental approach adopted as they well reproduce in mice the “earned security” phenomenon described in humans. Further to this, our electrophysiological data point to the mesocorticolimbic DA system, and the DA neurons of the VTA in particular, as (one of the) neuronal network(s) playing a key role in mediating the long-lasting dysfunctional effects of adverse experiences during early phases of brain’s emotional/motivational development. More specifically, this study offers three main findings. First, our analysis of a non-clinical population confirmed previous findings obtained in clinically depressed subjects^3^^,^^5^ by indicating that women with the highest score for secure attachment show the lowest score for depression symptoms in adult life. Moreover, our data revealed that in sharp contrast with findings obtained in clinically depressed patients,^36^ in non-clinical women, the experience of early traumas does not necessarily predict the severity of depressive symptoms. This finding does not support the requirement of traumatic experiences for disruption of secure attachment while supporting the view that early alteration of attachment bond represents a condition of fragility toward depressive symptoms fostered by subsequent adversities.^37^ Attachment patterns established during infancy are based on internal working models of the attachment figure and the self^38^ and typically persist during adulthood^2^^,^^38^^,^^39^ influencing future behaviors and relations. However*,* as interpersonal emotional relations mature and/or change over the years,^40^^,^^41^^,^^42^^,^^43^ the adult attachment style does not necessarily reflect the childhood one,^42^^,^^44^^,^^45^ as the internal working models may also change following later events.^46^ Although relatively stable, the attachment pattern could, indeed, be influenced by significant experiences (i.e., stress exposure, trauma, and romantic relationship) that can lead to adjustments or changes in adulthood.^41^^,^^45^^,^^47^^,^^48^^,^^49^ Longitudinal studies evaluating the attachment style in different life phases would let us understand whether the early attachment patterns are maintained in adulthood.

Second, our mouse model revealed that DBA female pups exposed to RCF develop an altered attachment bond with the caregiver and altered activity of VTA DA neurons. In contrast, RCF does not affect the attachment bond in DBA male pups, as evaluated by MSS. This is consistent with previous results evidencing how exposure to RCF affects adult DBA mice’s depressive-like behaviors (i.e., FST and TST) and related transcriptional profile within VTA selectively in female mice, with no significant effects in DBA males.^15^^,^^22^ Third, our findings demonstrate that the presence of a stable significant caregiver (SAC) during the RCF procedure is sufficient to promote the development of a secure attachment with the last foster mother and its ability to moderate pups’ emotional reactivity. Moreover, this procedure also prevented the RCF-induced expression of depressive-like behavior and alteration of VTA DA neuronal activity in adulthood.

Together, our data strongly support translational value of the mouse model adopted and confirm VTA DA neurons as relevant players during the neurodevelopmental processes influenced by the early newborn-caregiver relationship.

### A mouse model of “earned-secure attachment”: Insight for clinical research on depression

Human studies indicate that the availability, during childhood, of a significant alternative attachment figure providing stability and emotional support can help to prevent the adverse outcomes of primary insecure attachment in adult life.^8^^,^^9^ Our findings in mice are in line with these data. Thus, stepping from our knowledge of the RCF protocol to model insecure attachment,^13^^,^^15^^,^^19^^,^^20^^,^^21^^,^^22^^,^^23^ we sought to set up a mouse model of earned-secure attachment by including the equivalent of the significant alternative attachment figure in humans, a SAC, during the RCF. We adopted the rodent strange situation paradigm^35^ to evaluate how early experiences may disrupt the attachment-like style in mice and, importantly, how an alternative, significant caregiver could counteract such alteration.

The most relevant index of children’s secure attachment is their seeking for reassurance from their caregivers during reunions following temporary separations.^34^^,^^50^^,^^51^ This response is modeled in rodent pups by the increased proximity to the caregiver upon the reunion following a brief separation.^35^^,^^51^ Such phenotype was absent in RCF-exposed females but still significant in RCF-exposed males and RCF-exposed females in the presence of a stable alternative attachment figure during RCF. These results demonstrated that while indeed DBA RCF female pups showed an insecure attachment-like style, the presence of the SAC prevented the attachment alteration by providing a stable, secure base. The protective effect of the SAC was confirmed by our evaluation of separation anxiety with USVs analysis.^52^ Indeed, analogously to previous data for outbred and inbred mouse strains as well as rats, RCF exposure enhanced separation anxiety,^16^^,^^18^^,^^22^ and, notably, this effect was counteracted by SAC exposure.

It is essential to point out that the foster mothers did not show reduced or disruptive maternal care to the pups, thus supporting the conclusion that severe adverse experiences, such as parental neglect or abuse, are not necessarily required to affect secure attachment. On the other hand, there is compelling evidence that unpredictable signals by caregivers during a sensitive phase of development can negatively influence mammals’ neurodevelopment,^53^ including the maturation of the brain’s emotional and motivational systems,^54^ such as the mesocorticolimbic circuit.^15^^,^^19^^,^^20^^,^^21^^,^^22^ Indeed, the behavior of five different caregivers (the biological mother on the first day of life and the foster mothers on the following four days) can undoubtedly be challenging to become predictable for the developing pups.

Crucially, the SAC protective effects were not limited to early life; the presence of SAC also prevented the development of a depression-like phenotype consistently reported in adult RCF DBA females.^15^^,^^19^^,^^22^^,^^23^ Together, our behavioral data confirmed that the presence of a SAC during the RCF promoted secure attachment and prevented the long-lasting consequences of early repeated adoptions on adult depression-like behavior in females.

### I_h_ current in iVTA DA neurons represents one of the possible mediators of depression-like phenotype related to dysfunctional attachment in mouse

Following, to investigate a possible causal link between early attachment and dysfunctional phenotypes in our animal model, we measured the I_h_ current in DA neurons located in the “intermediate” VTA (defined as in Krashia et al.^55^). VTA DA neurons project to several brain areas within the mesocorticolimbic circuit and are deeply involved both in motivation and depression^56^^,^^57^^,^^58^^,^^59^ and in attachment bond formation.^2^^,^^60^^,^^61^^,^^62^ The DA mesolimbic pathway is mainly sensitive to stress and particularly to early life experiences, both in rodents and humans.^22^^,^^23^^,^^36^^,^^63^^,^^64^^,^^65^^,^^66^^,^^67^^,^^68^^,^^69^^,^^70^ For instance, in mouse models, RCF females of DBA or C57BL/6J strains, respectively characterized by depressive-like or resilient-like phenotypes in adulthood, show enhanced or decreased mesocortical dopamine release in response to an acute stress challenge.^19^ Analogously, VTA adaptations have been reported in depressed patients.^58^^,^^59^^,^^71^^,^^72^^,^^73^^,^^74^

The hyperpolarization-activated current (I_h_ current) is considered a functional hallmark of VTA DA neurons.^55^^,^^75^^,^^76^ *In vivo* inhibition of the I_h_ currents in VTA DA neurons was shown to mimic the effects of antidepressants in animal models,^23^^,^^77^ and RCF-exposed adult females of the C57BL/6J inbred strain, which are resilient to the RCF pro-depressant effects, are characterized by reduced I_h_ current in DA neurons of the iVTA.^23^ Therefore, here we tested the hypothesis that RCF-exposed individuals of the DBA strain, genetically susceptible to developing depressive-like phenotypes in adulthood, could be characterized by increased I_h_ current in iVTA DA neurons. Our results confirmed this hypothesis and suggested a relationship between the functional, long-term modulation of this cellular type and the development of insecure attachment with the caregiver. Notably, the presence of the SAC during the first days of postnatal life prevented the RCF-induced increase of I_h_ current in iVTA DA neurons.

Thus, our findings confirm a significant role for altered maturation of elements within the mesocortical DA system in females’ risk of developing depressive-like phenotypes in adulthood, and in particular point to DA VTA neurons. VTA DA neurons are the primary source of DA both for the mesocortical circuit (targeting the medial pre-frontal cortex [mpFC] in rodents) and for the mesolimbic systems (mainly nucleus accumbens and amygdala).^77^^,^^78^^,^^79^^,^^80^^,^^81^ While the involvement of the VTA-amygdala DA transmission in depressive phenotypes has been challenged,^80^ there is strong evidence for a role of the DA transmission onto mpFC in depression.^82^^,^^83^^,^^84^ Interestingly, in mice, DA innervation of the rodent mpFC starts between postnatal days 2 and 4,^85^ a time window coinciding with the application period of the RCF protocol. Analogously, in humans, the alteration of the child-parent relationship (between 0 and 24 months) is particularly detrimental to corticolimbic maturation^86^^,^ and early childhood stress alters the development of VTA-mpFC connectivity.^87^ Thus, mpFC represents a brain area definitively worth investigating with help from animal models when addressing the link between early attachment and dysfunctional phenotypes in adulthood. It is known that distinct nuclei of the VTA do project to different brain areas, from which they also receive feedback innervation.^88^ Thus, it seems more than plausible that DA neurons of neighboring regions *within* the VTA may regulate differently depression-related neural circuits and behaviors.^89^^,^^90^^,^^91^ For instance, Zhong et al.^71^ reported reduced activity of DA neurons of the lateral parabrachial nucleus of the VTA (projecting mostly to the lateral shell of the NAc) in mice following chronic stress. Analogously, an innervation target-related modulation of the I_h_ current was present in distinct populations of VTA DA neurons from mice following chronic social defeat.^92^ Thus, RCF might induce in DBA RCF females a long-term modulation of the I_h_ current in DA neurons of the medial or lateral VTA distinct from what we observed in the iVTA. These aspects are currently under investigation in our laboratory.

Insofar only a few attachment-based interventions were shown to reduce rates of insecure attachment in early childhood^5^^,^^93^^,^ and no study examined whether modifications to the attachment style can reduce the risk for depression in adulthood, with the relevant underlying mechanisms staying unknown.

By proposing the animal model of earned-secure attachment, we demonstrate that the presence of a SAC is sufficient to prevent the short-term and long-lasting behavioral and electrophysiological alterations induced by unstable attachment in mice and provide a tool to address this topic experimentally.

### Limitations of the study

With regard to the analysis of human data reported in this study, the interpretation of the results is limited by the use of a single time point for measures and retrospective assessments. Specifically, the attachment style is assessed by a retrospective self-report questionnaire. However, since the attachment pattern is affected by many factors throughout life, the adult style does not necessarily reflect the childhood one. Longitudinal studies with multiple time points and clinical interviews could provide more detailed information to better understand whether early attachment patterns are maintained in adulthood. Moreover, as reported in the results section, men and women show different predictors of depressive symptoms, though the relatively small number of men in our sample might hinder the generalization of our results to the male population. Also, the small variation of age in our sample prevents a proper generalization to the general population.

Regarding adopting animal models to mimic human behavior, this approach is inevitably prone to be affected by some levels of “simplification”; we are aware of this limitation. However, the attachment bond is a phylogenetically preserved behavioral system,^2^^,^^11^^,^^12^^,^^94^ and exploiting and further implementing our well-validated mouse model of early adversity allowed us to go beyond the correlational limitations intrinsic in human studies. Here, by proposing a rodent model of earned-secure attachment and by indicating the modulation of the I_h_ current in DA iVTA neurons as a potential marker at the cellular level, our results shed light on the biological mechanisms linking attachment and depression.

## Resource availability

### Lead contact

Further information and requests for resources and reagents should be directed to and will be fulfilled by the lead contact, Rossella Ventura (rossella.ventura@uniroma1.it).

### Materials availability

This study did not generate new unique reagents.

### Data and code availability

• This paper reports an original code (supplementary information).
• Data reported in this paper will be shared by the lead contact upon request.
• Any additional information required to reanalyze the data reported in this paper is available from the lead contact upon request.

## Acknowledgments

This work was supported by Ateneo Sapienza, Rome, Italy, 2020 (RM120172B7A3A801), 2021 (RG12117A5C2F8800), 2021 (RP12117A58C6D266), and 2023 (RP123188ED6560C7). We also acknowledge a contribution from the Italian National Recovery and Resilience Plan (NRRP), M4C2, funded by the 10.13039/501100000780European Union – NextGenerationEU (Project IR0000011, CUP B51E22000150006, “EBRAINS-Italy”). All images have been prepared with BioRender.com (NM25YJXUGE). We thank Dr. Francesca R D’Amato for her useful suggestions.

## Author contributions

Conceptualization of animal study, investigation, methodology: C.M. and L.B.; investigation (animal model): M.D.S., S.L.D., A.P., and D.M.; formal analysis: F.F. and M.I.; writing – review & editing: D.A., C.C., and S.C.; investigation, formal analysis (animal model): G.C. and E.S.; conceptualization, formal analysis, writing – original draft, writing – review & editing, resources: M.R.; conceptualization of human and animal study, writing – original draft, writing – review & editing, resources, project administration: R.V. All authors have read and agreed with the final version of the manuscript.

## Declaration of interests

The authors declare no competing interests.

## STAR★Methods

### Key resources table

**Table:** 

REAGENT or RESOURCE	SOURCE	IDENTIFIER
**Antibodies**		

Donkey 488	Life Technologies	cat#A21202; RRID: AB_141607
TH antibody	Altlas Antibodies	cat#AMAb91112; RRID: AB_141607
Streptavidin 555	Invitrogen	cat#S32355; RRID: AB_2571525
Biocytin	Sigma-Aldrich	cat#B4261; CAS:576-19-2
Biocytin	Tocris	Cat# 18075902; CAS: 576-19-2

**Chemicals, peptides, and recombinant proteins**		

Triton X-	Merck	1086431000
Fluoromont	Merck	F4680
NMDG	Sigma-Aldrich	M2004
KCl	Sigma-Aldrich	P9333
NaH2PO4	Sigma-Aldrich	S9638
NaHCO3	Sigma-Aldrich	S5761
HEPES	Sigma-Aldrich	H4034
Glucose	Fisher Scientific	G/0450/60
Na-ascorbate	Sigma-Aldrich	A4034
Na-pyruvate	Sigma-Aldrich	P2256
CaCl2	Sigma-Aldrich	C3881
MgSO4	Sigma-Aldrich	M2773
NaCl	Sigma-Aldrich	S7653
Thiourea	Sigma-Aldrich	T7875
K-gluconate	Sigma-Aldrich	P1847
MgCl2	Sigma-Aldrich	M2393
ATP-Mg2	Sigma-Aldrich	A9187
GTP-Na3	Sigma-Aldrich	G8877
Phosphocreatine-Na2	Sigma-Aldrich	P7936
EGTA	Sigma-Aldrich	E4378

**Deposited data**		

Analyzed data	This paper	

**Experimental models: Organisms/strains**		

Mouse: DBA/2J	Charles River	#625

**Software and algorithms**		

IBM SPSS Statistics for Windows, Version 23.0	IBM Corp (Armonk, NY)	N/A
pClamp9	Molecular Devices	https://www.moleculardevices.com/
IGOR Pro	Wavemetrics	https://www.wavemetrics.com/
NeuroMatic	https://doi.org/10.3389/fninf.2018.00014	http://www.neuromatic.thinkrandom.com/
Prism	GraphPad	

### Experimental model and study participant details

#### Human study

We started investigating the possible link between attachment style and depressive symptoms in a nonclinical population (Figure 1A). Lime survey was used to collect surveys from 901 people, and the following exclusion criteria were applied: incomplete survey with missing data (*n* = 389). Five hundred twelve participants aged 18 years or older (398 women, age: 25,54 ± 0.03; 114 men, age: 26,14 ± 0.06 years) recruited from Sapienza University and Tor Vergata University (Rome) through word of mouth and flyers, were involved in the final analysis. Before enrollment, all participants were given a complete description of the study and signed a written informed consent (Prot. n. 0000268; 17/02/2022). The sample was composed of individuals allocated as a function of the Attachment scale (RQ; High or Low SA) and depression symptoms severity (PHQ-9 score; from Absent to Severe depression symptoms).

The study was approved by the Department of Psychology, Sapienza University Rome, Ethical Committee and complies with Helsinki’s declaration.

Parental care experienced in childhood was evaluated retrospectively using the Parental Bonding Inventory (PBI; 50 items assessing maternal and paternal care, with four subscales: 1. Maternal care, 2. Paternal care, 3. Maternal overprotection, 4. Paternal overprotection).^29^ Exposure to childhood trauma was measured using the short version of CTQ- Short Form (CTQ-SF; 28 items combined in 5 subscales: 1. Emotional abuse, 2. Physical abuse, 3. Sexual abuse; 4. Emotional neglect and 5. Physical neglect).^31^ The vulnerability to depressive symptoms was estimated using the Patient Health Questionnaire-9 (PHQ-9; self-report scale: 0–4, Absent; 5–9, Minimal; 10–14, Mild; 15–19, Moderate; >20, Severe).^32^ Last, the Relationship Questionnaire (RQ; 4 items^30^ was used to evaluate the attachment quality: SA, FA, PA, DA.

##### Artificial Neural Network Analysis

A simplified version of the ARIANNA model (ARtificial Intelligent Assistant for Neural Network Analysis) was adopted for the Artificial Neural Network analysis.^95^ ARIANNA is based on a Multilayer Perceptron Procedure and formed by 12 input layers (one for each independent variable), one hidden layer of five elements (instead of the two hidden layers of the original model), and a final output layer (depression). The architecture of the ANN was that of a FeedForward Neural Network (FFNN) with a hyperbolic tangent used as an activation function for all the units in all the layers, with data moving in only one direction from the input nodes through the two hidden layers to the output nodes. The chosen computational procedure was based on online training (details: initial learning = 1.2; lower learning = 0.001, learning epochs = 10, momentum = 0.9 interval center = 0, interval offset = ±0.5, mem size = 1,000, steps without error = 1, error change = 0.0001, error ratio = 0.001),with a partition between training and test of 70% and 30%. See supplementary information for details (Table S6).

#### Animal model study

##### Animals

6-8 weeks old DBA/2J (DBA) female and male mice (purchased from Charles River Laboratories, Italy) were housed in standard conditions with water and food available *ad libitum*, at constant room temperature (21 ± 1°C) and in a 12:12 h light-dark cycle (lights on at 07:00 a.m.).^15^^,^^19^^,^^22^^,^^23^ At 12 weeks old, DBA female and male mice were mated.^19^ Briefly, two females and one male were housed in transparent polysulfone cages with water and food *ad libitum*. After 15 days, pregnant females were isolated in clean cages with nesting material and inspected twice daily. Adequate measures were taken to minimize pain or discomfort of mice. All experiments were carried out in accordance with Italian national laws on the use of experimental animals (DL116/92 and DL26/2014; Experimental Protocol 901/2023 approved by the Italian Ministry of Health), in line with the European Communities Council Directives (86/609/EEC and 2010/63/UE).

### Method details

#### Repeated cross fostering

The RCF manipulation was performed as previously described^15^^,^^16^^,^^19^^,^^21^^,^^22^^,^^23^ (Figure 2A). Briefly, DBA pups from the same litter spent the first postnatal day (P0) with their biological mother. On P1, litters were randomly assigned to experimental (RCF) or Control (Cont) conditions. RCF pups were fostered (daily until P4, between 10:00 and 10.30 a.m.) by moving the entire litter from the biological to the AM’s cage. Pups were left with the last AM until weaning. Cont pups were only picked up daily and reintroduced in their home cage within 30 s. Animals were weaned at P28, sorted by sex, and housed in groups of 4 littermates. To avoid litter effects, RCF and Cont groups were sorted by collecting a maximum of 4 individuals per litter.^19^^,^^21^

#### Repeated cross fostering with significant attachment caregiver

The RCF with the Significant Attachment Caregiver (RCF+SAC) (Figure 2B) was as follows: 15 days after mating, DBA pregnant females were housed in clean cages with nesting material and a virgin non-lactating dam (SAC). Pups spent the P0 with the biological mother and the SAC (RCF+SAC). From P1, RCF+SAC pups were fostered by gently moving them with the assigned SAC into the home cage of a different AM, whose pups, with their respective SAC, were previously transferred to another AM. As for RCF, this procedure was repeated daily from P1 to P4; on P4, the SAC was removed, and the pups were left with the last AM until weaning.

#### Assessment of attachment-like style and maternal behavior evaluation

##### Mouse strange situation

To evaluate the attachment-like style, female and male pups (P18, female: 9–13, males: 6–7 mice) were tested in the MSS procedure (MSS) according to Lassi and Tucci^35^(Figure 2C). This procedure consisted of a 15-min pre-test phase and three experimental ‘sessions’ of 3 min each. During the pre-test phase, the mother (M) was placed with a genotype and age-matched virgin female (stranger, S) in a gray arena (60 × 60 × 60cm^3^) and left free to explore. For Cont mice, the mother (M) was represented by their biological mother, while for RCF and RCF+SAC mice, she consisted of the last AM. After the M-S familiarization, the pup was introduced to the arena and tested in three consecutive experimental sessions (1–3).

In the first one, the pup was in the arena with the mother (M1) and the stranger (S1). In the second session, the mother was removed from the arena, and the pup was left alone with the stranger (S2). In the third one, the mother (M3) returned to the arena with the pup and the stranger (S3). All MSS sessions were video recorded by a webcam and scored manually by a trained observer for each episode/mouse/litter.

For each session, we quantified the time (seconds) spent by the pup actively exploring (sniffing and touching) the Stranger (S1-3) and the Mother (M1/3)^35^ and the SAC (SAC1/3) for the RCF+SAC group. Three behavioral responses were evaluated as an index of attachment-like style: (1) the ‘Maternal Preference’ (comparison between M3 and S3); (2) the ‘Reunion’ (comparison between M3 and M1); and (3) the ‘Stranger Effect’, (comparison among S2, S1, and S3). Pups of each group (Cont, RCF, RCF+SAC) belonged to 4–5 litters.^17^

##### Ultrasonic vocalizations

Ultrasonic vocalization calls (USVs) were measured on P8 (8–12 female pups) during the separation from the mother (biological mother for Cont mice; last AM for RCF and RCF+SAC groups), as previously described.^2^^,^^16^ Each pup was individually placed into a sterilized beaker containing home-cage bedding (Home-cage), clean bedding (Clean), or SAC bedding (SAC) materials for the RCF+SAC group only. Vocalizations (number) were recorded for 5 min with an UltraSoundGate microphone (CM16, AvisoftBioacoustics, Berlin, Germany) placed 1 cm above the beaker and analyzed with dedicated software (Avisoft Bioacoustics, Berlin, Germany). Pups of each group (Cont, RCF, RCF+SAC) belonged to 4–5 litters.^17^

##### Maternal behavior

The maternal behavior (8–12 mice) observation was conducted as previously described.^16^ The observation was performed daily from P1 to P7, twice a day (from 12:00 to 12:30 and from 16:00 to 16:30), by an instantaneous sampling method (2 min of sampling/rate) for 16 sampling points for each session. The Nursing (N) and Grooming/Liking (G/L) behaviors shown by the biological mother (for Cont groups), AM (for RCF and RCF+SAC groups), and SAC (for RCF+SAC groups) were analyzed.

#### Assessment of depression and anxiety-like phenotype in adult mice

FST, TST, and OF were conducted in adulthood as previously described^19^^,^^20^^,^^22^ to evaluate depressive-like, locomotor activity, and anxiety-like behaviors. 6–8 animals were used for adult behavioral tests (P80-90, Figure 3A).

##### Forced swimming test

In FST, female mice were individually introduced for 10 min into a glass cylinder (height 40 cm, diameter 18 cm), filled with water (up to 20 cm; 28 ± 2°C), and the behavioral response was recorded for 10 min using a camera placed in front of the cylinder. The duration (seconds) of immobility (absence of movement) was manually scored with “Boris” Software by a trained observer blinded to the animals’ treatment.^21^^,^^22^^,^^96^

##### Tail suspension test

During TST, female mice were suspended by their tails using adhesive tape (1 cm from the top of their tails) at a height of 60 cm from the apparatus base.^15^^,^^97^ Mice behavior was video recorded for 10 min, and the duration of immobility (the period when the animals stopped struggling for ≥1 s) was manually scored by “Boris” software by a trained observer blinded to the animals’ treatment.^96^

##### Open field test

In OF, female mice were individually introduced in a circular apparatus of 60 cm in diameter and 20 cm in height. The distance moved (cm) and time spent (second) in the external part of the apparatus (percentage of total time spent in exploration) were recorded for 5 min and analyzed by the fully automated tracking video system “EthoVision” (Noldus, The Netherlands).^22^

##### Electrophysiology

Horizontal midbrain slices containing the VTA (260μm) were prepared as previously reported.^23^^,^^98^ Following no-return deep anesthesia with halothane, adult (P58–90) DBA female mice previously exposed to RCF, RCF+SAC or control manipulations were decapitated. Brains were rapidly dissected and moved to the vibratome in cold (0.5°C–4°C), 95%O_2_–5%CO_2_ –saturated N-methyl-D-glucamine (NMDG)-based ‘slicing’ solution (below); after cutting, slices underwent a temperature boost (∼34°C) during the progressive increase of extracellular Na^+^.^23^ For long-term storage, slices were transferred into a HEPES-containing, Na-based aCSF (below)^23^ and let recover for at least 1 h at room temperature (∼24°C) before patch-clamp experiments.

The N-methyl-D-glucamine (NMDG)-based ‘slicing’ solution contained, in mM: 92 NMDG, 2.5 KCl, 1.25 NaH_2_PO_4_, 30 NaHCO_3_, 20 HEPES, 25 glucose, 2 thiourea, 5 Na-ascorbate, 3 Na-pyruvate, 0.5 CaCl_2_, and 10 MgSO_4_ (pH to 7.3–7.4 with hydrochloric acid). The HEPES-containing, Na-based long-term storage aCSF contained (in mM): 92 NaCl, 2.5 KCl, 1.25 NaH_2_PO_4_, 30 NaHCO_3_, 20 HEPES, 25 glucose, 2 thiourea, 5 Na-ascorbate, 3 Na-pyruvate, 2 CaCl_2_, and 2 MgSO_4_ (95%O_2_–5%CO_2_; pH 7.3–7.4). For patch-clamp recordings, slices were moved to the recording chamber of an upright microscope (DMLSF; Leica) and continuously perfused (3–4 mL/min) with standard aCSF containing (in mM): 126 NaCl, 24 NaHCO_3_, 10 glucose, 2.5 KCl, 2.4 CaCl_2_, 1.2 NaH_2_PO_4_ and 1.2 MgCl_2_, saturated with 95%O_2_–5%CO_2_ (pH 7.4; ∼290 mOsm). All recordings were performed at near-physiological temperature (32°C–34°C), with no drugs added to the aCSF and no liquid junction potential correction. Recording pipettes were filled with a standard 125 K-gluconate-based ‘intracellular’ solution containing (in mM): 125 K-gluconate, 10 KCl, 10 HEPES, 2 MgCl_2_, 4 ATP-Mg_2_, 0.3 GTP-Na_3_, 0.75 EGTA, 0.1 CaCl_2_, 10 Phosphocreatine-Na_2_ (pH 7.2, ∼280 mOsm). For *post-hoc* immunofluorescence identification, the pipette solution was added daily with freshly weighted biocytin (0.2–0.4%). Cell-attached and whole-cell patch-clamp recordings were performed from visually identified (40×) VTA DA neurons (MultiClamp 700B and Digidata 1322A; Molecular Devices). Only neurons matching the following criteria were selected for patching and statistical analysis^55^: location in the intermediate region of the VTA (iVTA); presence of low-frequency, spontaneous action potentials (APs) in cell-attached and whole-cell configuration; presence of *I*_*h*_ current; positive *post-hoc* Tyrosine Hydroxylase (TH) immunostaining (Figure 3G). Spontaneous firing was recorded in cell-attached and in whole-cell configuration, immediately after membrane rupture (holding potential, V_H_ −60mV; *f*_*c*_ 10kHz; sampling 50kHz). The neuron approximates Resting Potential and intrinsic properties (membrane resistance, Rm; membrane time constant tau; and membrane capacitance, Cm) were also estimated soon after accessing the cell interior using the amplifier inbuilt voltmeters (30ms-long, +20mV voltage steps at 20Hz from V_H_ −60mV). In voltage-clamp, the hyperpolarization-activated inward current I_h_ was recorded in whole-cell configuration in response to hyperpolarizing voltage steps (1sec-long; from −60 to −120mV, 20mV increment, V_H_ –60 mV; *f*_*c*_ 2kHz; sampling 10kHz). The amplitude of I_h_ current was measured at the current steady-state and after nulling the current baseline at the interception between the offset of the capacitive peak and the onset of the hyperpolarization-activated inward current for each step response. These amplitude values were thus plotted against the voltage command in the ‘I–V curve.’ Cell excitability was investigated in current-clamp (fc 10kHz; sampling 50kHz; membrane potential held at approx. −60mV via steady current injection) by recording spontaneous and evoked AP firing and estimating the rheobase. The rheobase (the amplitude of the injected current necessary to induce the first AP) was obtained from the cell response to a series of 5pA-incremental consecutive steps of depolarizing injection current (I_inj_; 50ms-long steps; amplitude range: 0–0.2nA). Patch-clamp recordings were analyzed using Clampfit9 (Molecular Devices) and Igor. Pro 6.32A (WaveMetrics Inc) with NeuroMatic 2.8.^99^

##### Immunofluorescence

Immunofluorescence for TH labeling analysis was performed as previously reported.^23^ Patched brain slices were washed in PBS (room temperature), fixed overnight at 4°C in 4% paraformaldehyde solution (in 0.1M, pH 7.4; PBS), and washed before further processing (3×, 20′ each, PBS, r.t.). For TH labeling, slices were incubated with a TH primary antibody (1:500, 48h 4°C; Atlas Antibodies AMAb91112) in PBS containing 0.2% Triton X-100 (Merck 1086431000**;** PBT-X). Incubation with Streptavidin conjugate (1:400 in PBT-X, 2h r.t.; Alexa Fluor 555, Invitrogen S32355) and donkey anti-mouse secondary serum (1:200 in PBT-X, 2h r.t.; Alexa Fluor 488, Life Technologies A21202) followed by 3× PBS washes and slice mounting (Fluoromont; Merck F4680) allowed marking biocytin-filled and TH-positive neurons, respectively. Immunofluorescence images were acquired using a Nikon fluorescence microscope equipped with MetaMorph 7.6.5.0 (Molecular Devices).

### Statistical analysis

#### Human study

Comparisons between women and men for CTQ, PBI, RQ, and PHQ-9 were performed using Multivariate analysis of variance followed by Univariate analysis. Regression analysis (separately for women and men) was used to estimate the contribution of PBI, CTQ, and RQ on depressive symptoms. Statistical analyses were carried out with the help of SPSS for Mac, version 27.0. The Pearson coefficient was used to assess simple correlations. Multiple linear regression was used to identify the independent variables associated with the dependent variables. Coefficient B, t, and *p* values for this analysis are reported in Tables S2 e S3 on the Supplemental information. The adjusted coefficient of determination (R^2^) was used to assess the variance of the dependent variable explained by the independent ones. An Artificial Neural Network analysis was developed using the module Neural Networks of IBM SPSS Statistics for Windows, Version 23.0 (Armonk, NY: IBM Corp) according to a simplified version of the ARIANNA model already used in previous studies.^95^ The chosen computational procedure was based on online training, with a partition between training and test of 70% and 30%.

#### Animal model study

For behavioral data, statistical analysis was conducted by SuperANOVA Statistical Software, and we considered a statistically significant *p*-value <0.05. Data are presented as mean ± SEM.

For MSS, “Maternal Preference”, “Strange Effect”, and “Maternal Reunion” were analyzed by two-factor ANOVAs, separately for each sex, with time spent in interaction with mother and/or stranger in different sessions (for “Maternal Preference” 2 levels: M3, S3; for “Reunion” 2 levels: M1, M3; for “Strange effects” 3 levels: S1, S2, S3) as repeated measures and early life experience as an independent factor (treatment: 2 levels: RCF and Control). To investigate the rescue effects of SAC on these parameters in female pups, repeated measures ANOVAs were conducted to analyze the time spent interacting with the mother and stranger (For “Maternal Preference” 2 levels: M3, S3; for “Reunion” 2 levels: M1, M3; for “Strange effects” 3 levels: S1, S2, S3) and with the SAC and stranger (for “Maternal Preference” 2 levels: SAC3, S3; for “Reunion” 2 levels: SAC1, SAC3; for “Strange effects” 3 levels: S1, S2, S3).

The number of pups USVs emitted during separation from mother was investigated by Two-way ANOVAs (independent factors: treatment, 3 levels: Cont, RCF, RCF+SAC; and bedding, 2 levels: Clean, Home-cage). Moreover, the number of RCF-SAC USVs emitted in the presence of SAC bedding was analyzed by one-way ANOVA (independent factor bedding, 3 levels: Clean, Home-cage, SAC).

The frequency of maternal behaviors (Nursing and Grooming/Liking) from P1 to P7 was analyzed by one-way ANOVAs (independent factor: treatment, 3 levels: Cont, RCF, RCF+SAC). Moreover, to investigate the frequency of maternal behavior received by RCF+SAC pups from P1 to P4 from AM and SAC, Student t-tests separately for Nursing and Licking/Grooming (Nursing, Licking/Grooming) were performed.

To investigate the depression-like phenotype, the time spent in immobility (seconds) during FST and TST was analyzed by one-way ANOVAs (independent factor: treatment, 3 levels: Cont, RCF, RCF+SAC). The moved distance (during OF) and the anxiety-like behavior (as a percentage of time spent in the external area of OF) were analyzed by one-way ANOVAs (independent factor: treatment, 3 levels: Cont, RCF, RCF+SAC). Individual between-group comparisons were performed, when appropriate, by *post-hoc* analysis. For *ex vivo* (patch-clamp) experiments, statistical analysis was run in Prism 6 (GraphPad) using Mann–Whitney *U* test, unpaired Student’s *t* -t-tests (with Welch’s correction), one-way or two-way repeated measures ANOVA with Bonferroni’s *post-hoc* test or Kruskall-Wallis with Dunn’s *post-hoc* test, as required. The normality of datasets was evaluated using either the Shapiro–Wilk test or the D'Agostino & Pearson omnibus normality test (*p* < 0.05 was considered significant).

## Footnotes

Supplemental information can be found online at https://doi.org/10.1016/j.isci.2024.111328.
