# Supplementary material for: Secure attachment to caregiver prevents adult depressive symptoms in a sex-dependent manner: A translational study
Source: iScience. 2024 Nov 21;27(12):111328. doi: 10.1016/j.isci.2024.111328 (PMC11700650; doi:10.1016/j.isci.2024.111328)
Supplement: Document S1. Figures S1, S2, and Tables S1–S6 [file mmc1.pdf]

iScience, Volume *27*

Supplemental information

Secure attachment to caregiver prevents adult depressive symptoms in a sex-dependent manner: A translational study

Camilla Mancini, Lucy Babicola, Gilda Chila, Matteo Di Segni, Diana Municchi, Sebastian Luca D'Addario, Elena Spoleti, Alice Passeri, Carlo Cifani, Diego Andolina, Simona Cabib, Fabio Ferlazzo, Marco Iosa, Rodolfo Rossi, Giorgio Di Lorenzo, Massimiliano Renzi, and Rossella Ventura

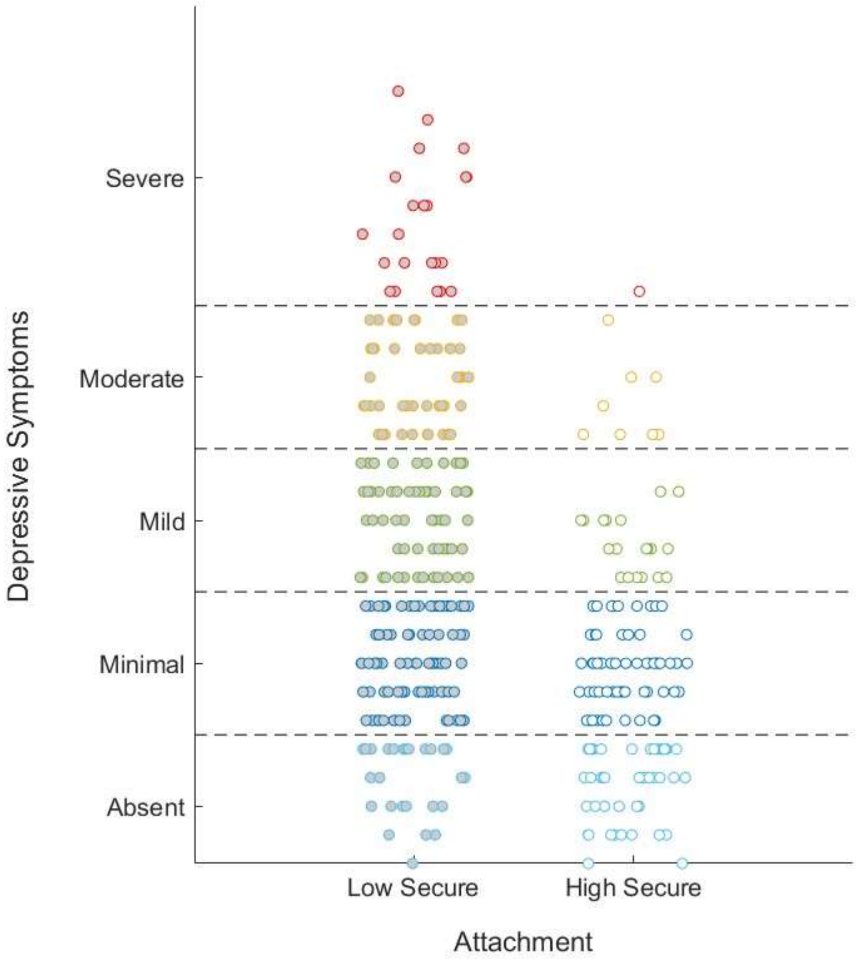

Figure S1: Scatter plot displaying the score for depressive symptoms related to secure attachment score (low/high secure attachment), related to Figure 1. Each dot represents a subject's PHQ-9 result.

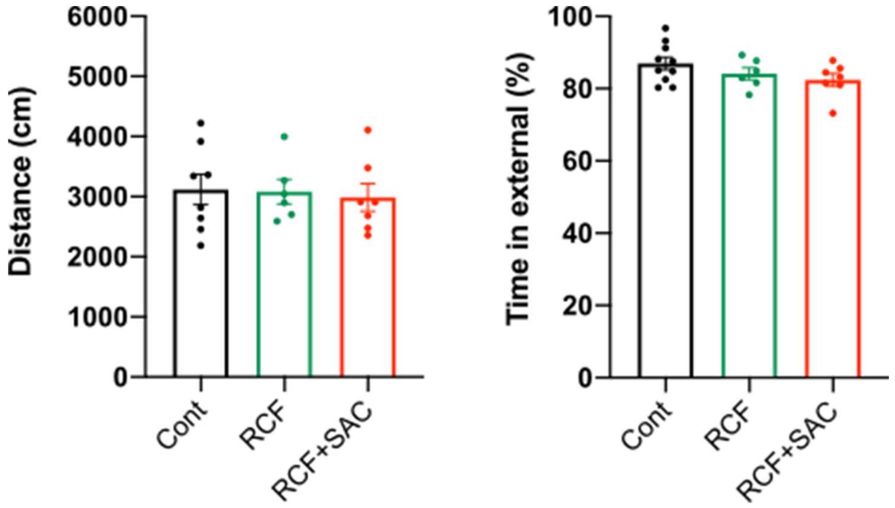

Figure S2. Moved distance (cm, left panel) and percentage of time spent in external part (%, right panel) during Open Field test, related to Figure 2.

	Women	Men	p
Trauma score	24.06 (18.02)	21.17 (16.46)	ns
Secure Attachment	4.09 (1.5)	4.17 (1.56)	ns
Dismissing Attachment	3.46 (1.87)	3.19 (1.81)	ns
Preoccupied Attachment	3.55 (2.01)	3.45 (2)	ns
Fearful Attachment	1.97 (1.33)	2.09 (1.28)	ns
Maternal Care	24.97 (8.22)	26.78 (8.06)	<0.05
Paternal Care	21.16 (8.95)	23.37 (7.9)	<0.05
Maternal Overprotection	13.73 (8.75)	14.34 (8.42)	ns
Paternal Overprotection	11.71 (8.02)	10.99 (6.8)	ns
PHQ-9	9.42 (5.61)	8.43 (5.31)	0.094

Table S1. Average scores and standard deviations (in parenthesis) for each questionnaire as a function of gender, related to Figure 1. p-values refer to the results of the univariate analyses of variance.

**Women-PHQ-9 test**	**B**	**t**	**p**
Secure Attachment	-0.887	-4.554	<0.001
Dismissing Attachment	0.633	3.946	<0.001
Preoccupied Attachment	0.527	3.898	<0.001
Fearful Attachment	0.177	0.930	ns
Maternal Care	0.018	0.522	ns
Paternal Care	-0.033	-1.034	ns
Maternal Overprotection	0.061	1.813	ns
Paternal Overprotection	0.013	0.374	ns
Total score Trauma	-0.015	-1.168	ns

Table S2. Regression analysis entering CTQ total score, related to Figure 1.

**Men-PHQ-9 test**	**B**	**t**	**p**
Secure Attachment	0.042	0.129	ns
Dismissing Attachment	0.663	2.294	p<0.05
Preoccupied Attachment	0.725	3.084	p<0.005
Fearful Attachment	0.273	0.844	ns
Maternal Care	-0.061	-0.989	ns
Paternal Care	-0.257	-4.284	p<0.001
Maternal Overprotection	-0.021	-0.305	ns
Paternal Overprotection	-0.046	-0.578	ns
Tot score Trauma	0.004	0.174	ns

Table S3. Regression analysis entering CTQ total score, related to Figure 1.

**Group** / ***Session***	**Cont Female**	**RCF Female**	**Cont Male**	**RCF Male**	**RCF+SAC Female**
Maternal Preference *(M3/SAC3vsS3)*	$p<0.001$ M3>S3	*n.s.* M3=S3	$p<0.001$ M3>S3	$p<0.05$ M3>S3	$p<0.05$ SAC3>S3
Reunion *(M3/SAC3vsM1/SAC1)*	$p<0.001$ M3>M1	*n.s.* M3=M1	$p<0.05$ M3>M1	$p<0.005$ M3>M1	$p<0.005$ SAC3>SAC1
Stranger Effect *(S1vsS2vsS3)*	$p<0.001$ S1<S2<S3	*n.s.* S1=S2=S3	$p<0.001$ S1<S2>S3	$p<0.005$ S1<S2>S3	$p<0.005$ S1<S2>S3

Table S4. Synoptic results of the MSS test for all the experimental groups (male and female RCF and Control mice; females RCF+SAC) in the different sessions (Maternal preference, Reunion, and Stranger Effect), related to Figure 2.

	**Cont**	**RCF**
Intrinsic properties		
**RP**<br>(mV)	– 48.1 ± 0.9 (17)	– 48.0 ± 2.0 (12)
**C_m**<br>(pF)	61 ± 6 (18)	61 ± 6 (11)
**R_m**<br>(MOhm)	115 ± 19 (7)	165 ± 14 (9)
**τ_{memb}**<br>(ms)	1.5 ± 0.2 (7)	1.4 ± 0.2 (9)
Cell excitability		
**Spontaneous firing**<br>(*cell-attached*)<br>(Hz)	3.0 ± 0.5 (12)	4.2 ± 1.3 (5)
**Spontaneous firing**<br>(*whole-cell*)<br>(Hz)	2.8 ± 0.5 (17)	4.1 ± 0.9 (10)
**Rheobase**<br>(pA)	103 ± 22 (13)	97 ± 27 (12)

Table S5. Intrinsic properties and excitability of DA neurons of the intermediate VTA, related to Figure 3. The table reports mean ± SEM values for each parameter. In parenthesis, the number of neurons analyzed. All comparisons resulted statistically *n.s.* indicating lack of long-lasting effects of the RCF protocol on the general electrophysiological properties of identified TH^+ neurons (statistical analysis as in Methods).

Code

```
*Multilayer Perceptron Network.
MLP Depressione_PunteggiototalePHQ9 (MLEVEL=S) BY Abusoemotivo Abusofisico
    Trascuratezzaemotiva Trascuratezzafisica Attaccamentosicuro
    Attaccamentodistanzianteevitante Attaccamentopreoccupato
Attaccamentopaurosoevitante
    ScalaAccudimentoMADRE ScalaAccudimentoPADRE
    ScalaIperprotettivitàMADRE ScalaIperprotettivitàPADRE
 /RESCALE DEPENDENT=STANDARDIZED
  /PARTITION  TRAINING=7  TESTING=3  HOLDOUT=0
  /ARCHITECTURE   AUTOMATIC=NO HIDDENLAYERS=1 (NUMUNITS=5)
HIDDENFUNCTION=TANH
    OUTPUTFUNCTION=IDENTITY
  /CRITERIA TRAINING=ONLINE OPTIMIZATION=GRADIENTDESCENT LEARNINGINITIAL= 0.4
LEARNINGLOWER= 0.001
    LEARNINGEPOCHS= 10 MOMENTUM= 0.9 INTERVALCENTER=0 INTERVALOFFSET=0.5
MEMSIZE=1000
  /PRINT CPS NETWORKINFO SUMMARY IMPORTANCE
  /PLOT NETWORK PREDICTED
  /STOPPINGRULES ERRORSTEPS= 1 (DATA=AUTO) TRAININGTIMER=ON (MAXTIME=15)
MAXEPOCHS=AUTO
    ERRORCHANGE=1.0E-4 ERRORRATIO=0.001
 /MISSING USERMISSING=EXCLUDE .
```

Tab. S6. As reported in our study: An Artificial Neural Network analysis was developed using the module Neural Networks of IBM SPSS Statistics for Windows, according to a simplified version of the ARIANNA model. The code in SPSS language is reported in the following paragraph when the target variable was the depression. The code works for the version 23.0 of SPSS (Armonk, NY: IBM Corp). Related to Fig.1.
